# Preeclampsia 2.0: limitations and challenges of the two-stage hypothesis, and beyond

**DOI:** 10.1093/molehr/gaag001

**Published:** 2026-01-06

**Authors:** Berthold Huppertz

**Affiliations:** Division of Cell Biology, Histology and Embryology, Gottfried Schatz Research Center, Medical University of Graz, Graz, Austria

**Keywords:** preeclampsia, diagnosis, two-stage hypothesis, limitations, cardiovascular, placental hypoxia, placental senescence

## Abstract

The pregnancy-specific syndrome preeclampsia remains the syndrome of hypotheses. Still, there is not a single hypothesis explaining the etiology of the full spectrum of preeclampsia. This has direct consequences for the clinical management of the syndrome. So far, no single early biomarker has been identified to predict all women who will develop preeclampsia later in pregnancy. Similarly, no preventive treatment for all types of preeclampsia has been integrated into clinical routine. Interestingly, the last decade has not seen much progress in the quest of identifying the pathophysiological processes resulting in the clinical syndrome preeclampsia. This could be due to the following: (i) the preeclampsia definition has been immensely altered and widened to include a large variety of clinical subgroups; and/or (ii) scientists and clinicians still adhere to the already challenged two-stage hypothesis and give only little room for new hypotheses. These two reasons could have thwarted the deciphering of the etiology of preeclampsia. This review will describe the limitations and challenges of the two-stage hypothesis. It will also highlight some of the new ideas and theories that have been put forward. In conclusion, there is an urgent need for new concepts that allow a better explanation of the diversity of preeclampsia regarding symptoms and time of occurrence. This in turn will result in more options to develop specific predictive biomarkers and personalized treatment options.

## Graphical abstract

**Figure:**
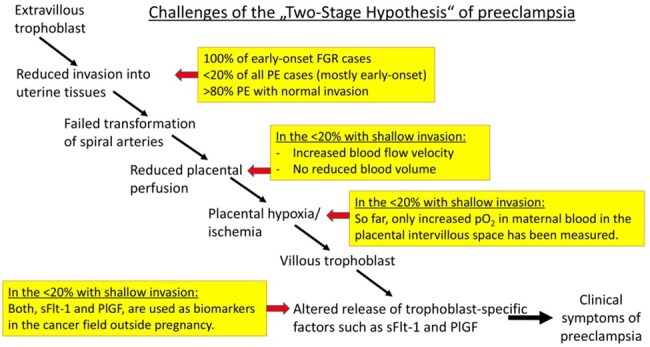
**Challenges of the ‘two-stage hypothesis’ of preeclampsia: each of the challenges (yellow boxes) of the two-stage hypothesis may result in its falsification.** FGR, fetal growth restriction; PE, preeclampsia; PlGF, placental growth factor; pO_2_, oxygen partial pressure; sFlt-1, soluble Fms-like tyrosine kinase-1.

## Introduction

The pregnancy-specific syndrome preeclampsia continues to be a major threat for pregnant women and their babies and has not lost any of its frightful symptoms and consequences. Especially the combination of early onset and severe symptoms immensely affects mothers and babies and results in increasing rates of maternal and newborn mortality and morbidity, even decades after pregnancy (Fingar *et al.*, 2017; Staff, 2019; Turbeville and Sasser, 2020). Particularly this combination poses a threat on mother and child as it is associated with lifelong negative effects for both individuals (Staff, 2019; Turbeville and Sasser, 2020). Unfortunately, the portion of severe preeclampsia in all preeclampsia cases is increasing, in the USA by 50% from 2005 to 2014 (Fingar *et al.*, 2017).

Due to the clinical importance of this pregnancy-specific syndrome, scientists and clinicians all over the world have been actively working for decades on elucidating the origin and etiology of preeclampsia. However, preeclampsia is still called the ‘syndrome of hypotheses’ as the initial event(s) and the subsequent pathophysiological alterations resulting in the clinical symptoms of preeclampsia are still unknown. This is why a multitude of hypotheses have been generated, challenged, falsified, and refreshed, but still not a single one can explain where and why this syndrome develops (Huppertz, 2008; Jung *et al.*, 2022).

Although the quest to identify the etiology of preeclampsia has not been successful so far, the massive efforts of the last decades have led to the identification of the first predictive biomarkers and therapeutic approaches, at least for a small subset of preeclampsia cases (Chaemsaithong *et al.*, 2022; Verlohren *et al.*, 2022). However, none of these efforts has resulted in the identification of a single, preeclampsia-specific biomarker for monitoring onset and progression of the syndrome (Rolnik *et al.*, 2024). The biomarkers currently in use to predict preeclampsia (PlGF and sFlt-1) are not specific for the syndrome and only predict a small percentage of cases (Huppertz, 2018b). Regarding the specificity of these markers for pregnancy/preeclampsia, it needs to be stressed that PlGF is used as a biomarker in a number of different cancer types such as colorectal and ovarian cancer (Wei *et al.*, 2005; Meng *et al.*, 2018), while sFlt-1 shows significant value in breast cancer and melanoma models (Verrax *et al.*, 2011; Smalley *et al.*, 2020). Hence, outside pregnancy, these (anti-) angiogenic factors are already used as biomarkers on a similar concentration level as during pregnancy (Huppertz, 2018b).

Regarding therapeutic options, the ASPRE study has shown that low dose aspirin given daily from 11 to 14 weeks of pregnancy to 36 weeks significantly lowers the rate of early-onset preeclampsia (Rolnik *et al.*, 2017, 2022). Luckily, the early-onset cases that show a beneficiary effect when treated with aspirin can be predicted by the current biomarkers PlGF and sFlt-1 (Chaemsaithong *et al.*, 2022). Unfortunately, this subtype only comprises about 10–20% of all preeclampsia cases (Robillard *et al.*, 2020).

This review will highlight the challenges and limitations of the two-stage hypothesis on the etiology of preeclampsia. It will also outline new approaches in explaining the onset of the syndrome. Finally, this review will hopefully aid the preeclampsia field to come back on track to finally reach the goal to identify the sources and pathways resulting in the diverse clinical symptoms of preeclampsia.

## Pitfalls in the definitions of preeclampsia and its subtypes

### Definition of preeclampsia subtypes

Due to the broad spectrum of symptoms and timelines related to preeclampsia, there are attempts to divide preeclampsia into smaller and better-defined subtypes. The two main attempts in place are (1) the division into ‘mild preeclampsia’ and ‘severe preeclampsia’ and (2) into ‘early-onset’ and ‘late-onset’ preeclampsia.

1. The first attempt has now been finished as there is the clear notion that a syndrome such as ‘mild preeclampsia’ does not exist. This is why the subtype ‘severe preeclampsia’ is no longer supported, and international societies such as the ISSHP (International Society for the Study of Hypertension in Pregnancy) only refer to ‘severe hypertension’ but no longer to severe preeclampsia (Magee *et al.*, 2022).
2. The second attempt is still active and many publications refer to the respective subtypes. Early-onset preeclampsia refers to preeclampsia before 34 weeks of gestation. Late-onset preeclampsia refers to preeclampsia after 34 weeks of gestation, with a subdivision of preterm preeclampsia (34–37 weeks) and term preeclampsia (after 37 weeks).

Although highly cited and used, the division into early- and late-onset displays a major pitfall as there are two different ways to divide preeclampsia into these two subtypes.

The first way uses the onset of symptoms before or after 34 weeks of gestation. If this way is used, the number of early-onset cases is massively increased, since a woman with diagnosis of preeclampsia due to first symptoms at 33 + 5 weeks may only deliver at 39 weeks, but is still grouped into the early-onset subtype as symptoms have started prior to 34 weeks. One recent example of this way of defining these subtypes is a study looking into the relation between preeclampsia subtypes and birth weight, analyzing more than 17 000 pregnant women. This study resulted in the following difference in gestational age at delivery: delivery in the early-onset group was 36.8 ± 3.5 weeks, delivery in the late-onset group was 39.0 ± 1.4 weeks (Ohseto *et al.*, 2025).

The second more stringent way uses the need for delivery before or after 34 weeks of gestation (Poon *et al.*, 2019). Here, the percentage of early-onset cases is less than 20% of all preeclampsia cases with most cases of preeclampsia delivering after 34 weeks of gestation (e.g. Robillard *et al.*, 2020).

This way of subdividing preeclampsia is related to another subdivision of preeclampsia. Early-onset preeclampsia is often related to ‘placental preeclampsia’ with high rates of fetal growth restriction (FGR) and reduced trophoblast invasion, while late-onset preeclampsia is often related to ‘maternal preeclampsia’ with higher rates of increased BMI of the pregnant women (Ness and Roberts, 1996; Robillard *et al.*, 2020).

Today, a simple literature subtype analysis of early-onset and late-onset preeclampsia is extremely difficult due to the varying definitions of the two subtypes. It would be extremely beneficial to agree on a single definition. Since the date of delivery is much easier to assess compared to the date of onset of symptoms, this author would recommend using delivery before and after the 34th week of pregnancy to separate early from late onset preeclampsia.

### Constantly changing definitions of how to diagnose preeclampsia

Ten years ago, the diagnosis of preeclampsia was based on hypertensive alterations of blood pressure in combination with kidney injury resulting in hypertension and proteinuria after 20 weeks of gestation in a pregnant woman with so far normal blood pressure (Townsend *et al.*, 2016). At the time, increased levels of systolic (≥140 mm Hg) and/or diastolic blood pressure (≥90 mm Hg) at any time during pregnancy were indicative for hypertension in pregnancy. In combination with proteinuria (≥300 mg protein/day in urine or an increased protein to creatinine ratio (≥3)) after 20 weeks of gestation, the diagnosis of preeclampsia was made (Table 1).

**Table 1.** Changes of the definition of preeclampsia during the last 10 years in German-speaking countries compared to other societies worldwide.

Parameter / Year	2014	2019	2024	2020	2022	2023
Area	German-speaking	German-speaking	German-speaking	ACOG	ISSHP	NICE
Chronic hypertension	*No* Only if superimposed with gestational hypertension	**Yes**	**Yes**	**Yes**	**Yes** Called superimposed preeclampsia on chronic hypertension	*No* Only if superimposed with gestational hypertension
Gestational hypertension	**Yes**	**Yes**	**Yes**	**Yes**	**Yes**	**Yes**
Kidney (proteinuria)	**Yes**	Maybe ^*^	Maybe ^*^	Maybe ^*^	Maybe ^*^	Maybe ^*^
Brain	*No*	Maybe ^*^	Maybe ^*^	Maybe ^*^	Maybe ^*^	Maybe ^*^
Liver	*No*	Maybe ^*^	Maybe ^*^	Maybe ^*^	Maybe ^*^	Maybe ^*^
Lungs	*No*	Maybe ^*^	Maybe ^*^	Maybe ^*^	Maybe ^*^	*No*
Hematological system	*No*	Maybe ^*^	Maybe ^*^	Maybe ^*^	Maybe ^*^	Maybe ^*^
Placenta—FGR	*No*	Maybe ^*^	Maybe ^*^	*No*	Maybe ^*^	Maybe ^*^
Gastrointestinal tract	*No*	*No*	Maybe ^*^	*No*	*No*	*No*
Placenta—SGA	*No*	*No*	Maybe ^*^	*No*	*No*	*No*
Angiogenic factors sFlt-1 and PlGF	*No*	Maybe ^*^	Maybe ^*^	*No*	*No*	*No*

Yes, mandatory part of the diagnosis; Maybe, may be included in the diagnosis, but not mandatory; No, not included in the diagnosis; *, one of these parameters needs to be present along with hypertension for the diagnosis of preeclampsia. ACOG, American College of Obstetricians and Gynecologists; FGR, fetal growth restriction; ISSHP, International Society for the Study of Hypertension in Pregnancy; NICE, National Institute for Health and Care Excellence; PlGF, placental growth factor; sFlt-1, soluble Fms-like tyrosine kinase-1; SGA, small for gestational age.

Similar to other countries worldwide, in German-speaking countries, the definition of preeclampsia clearly changed in 2019: (i) hypertension (>140/90 mmHg) was still the main driver, but now any hypertension (also chronic forms that developed prior to pregnancy) was included in the diagnosis with noneed of change in severity of increased blood pressure during pregnancy; (ii) proteinuria was no longer a mandatory part of the diagnosis; and (iii) newly developing organ dysfunction became the major second part of the diagnosis. Organs included were the kidneys (proteinuria), the hematological system (HELLP syndrome included), the liver (HELLP syndrome included), neurological abnormalities, the lungs, the placenta (FGR included), and ‘preeclampsia-specific systems’ (sFLt-1 and PlGF included) (Lapaire *et al.*, 2019) (Table 1). From now on, preeclampsia was no longer a maternal-only syndrome since FGR became part of the definition.

Recently in 2024, the definition of preeclampsia was again adapted in the German-speaking countries. Similar to the definition in 2019, today, any hypertension is the basis for the diagnosis of the syndrome. In addition, the newly developing organ manifestations remain the second part of the diagnosis and include the brain, the liver, the lungs, the kidneys, the gastrointestinal tract, the hematopoietic system, the placenta (this time with small for gestational age (SGA) fetuses and FGR) and the angiogenic factors sFlt-1 and PlGF (S2k-Leitlinie, 2024). There is expert consensus that a differentiation between mild and severe preeclampsia is no longer appropriate since a ‘mild preeclampsia’ does not exist. An excellent overview of the changes of international guidelines during the last decades has been given by Tanner *et al.*, (2022) and summarized in Table 1.

The changes of the preeclampsia definition may at least partly explain the massively increasing rates of preeclampsia. In the USA, March of Dimes has described that ‘In the USA, it [preeclampsia] affects about 1 in every 25 pregnancies.’ (March of Dimes, 2025). Fink *et al.*, (2023) show a similar number for the year 2008 with 1 in every 22.5 women. However, this ratio has changed from 1 in every 22.5 women in 2008 to a ratio of 1 in every 12 women in 2021 (Fink *et al.*, 2023). This is a nearly 2-fold increase in the number of women diagnosed with preeclampsia within the last 13 years in the USA. Of course, due to the changes in demographics, other risk factors such as increased age and weight of pregnant women add to the increase of pregnancy pathologies such as preeclampsia.

Although there is this immense increase in the number of women diagnosed with preeclampsia, the quest to identify the source(s) of the dysfunctions leading to preeclampsia is impeded at the same time. One reason may be the continuous change of the definition of the syndrome preeclampsia. Of course, there is no doubt that such a syndrome has to be defined and diagnosed above all based on clinical needs and maternal and neonatal health issues, but it definitively slows down the scientific endeavor on identifying the etiology of preeclampsia.

Another reason for the current hindrance in identifying the pathophysiology of preeclampsia is the missing creativity to identify more appropriate pathways leading to the syndrome. It seems to be easier, in terms of thinking and in terms of publishing, to simply follow the classical pathway, while new avenues of thinking are hard to publish. Hence, the changes of definition and the missing flexibility in creating new hypotheses during the last two decades have thwarted the speed of the scientific endeavor to identify the etiology of preeclampsia.

## Hypotheses on the etiology of preeclampsia

### The two-stage hypothesis: already challenged

The still most-cited hypothesis to describe the pathogenesis of preeclampsia is often called the ‘two-stage model’. It describes the development of the syndrome as originating from abnormal placentation (i.e. defective trophoblast invasion), termed stage one. This is described to subsequently result in reduced placental perfusion with maternal blood, followed by placental hypoxia/ischemia with the later release of anti-angiogenic factors into maternal blood. This then is believed to cause widespread endothelial dysfunction (stage two) and the clinical symptoms of preeclampsia (Burton *et al.*, 2019; Sugulle *et al.*, 2024). While this hypothesis might capture some core elements of the syndrome, several substantial challenges limit its capacity to fully explain the molecular mechanisms as well as the clinical and epidemiological diversity of preeclampsia (Table 2).

**Table 2.** Limitations of the two-stage hypothesis.

**Timing** (gestational weeks)	Two-stage hypothesis	Limitations
Weeks 4–12	**Stage 1-1:** Reduced trophoblast invasion results in failure to transform spiral arteries	Present in <20% of all preeclampsia cases, mostly in early-onset cases; hence, cannot explain the etiology of >80% of all preeclampsia cases Present in nearly all early-onset, normotensive FGR cases Thus, this scenario is neither specific for preeclampsia nor can it explain the etiology of the majority of preeclampsia cases.
Week 12-delivery	**Stage 1-2:** Failure to transform spiral arteries results in reduced perfusion of the placenta with maternal blood	Blood volume per time interval very similar between controls and preeclampsia with reduced invasion Blood flow velocity into the placenta increases about 20-fold in the presence of reduced invasion, true for early-onset FGR and early-onset preeclampsia Thus, so far, no data for reduced perfusion exists, but calculations of increased flow velocity have been published, resulting in damage of villous tissues in early-onset FGR and early-onset preeclampsia.
Week 12-delivery	**Stage 1-3:** Reduced placental perfusion with maternal blood results in placental hypoxia/ischemia	A reduced pO_2_ in maternal blood in the intervillous space in preeclampsia has never been measured *in vivo* so far. By contrast, higher pO_2_ levels have been measured in cases with reduced trophoblast invasion, i.e. early-onset PE/FGR/PE+FGR. Thus, there is no *in vivo* data showing reduced pO_2_ levels in maternal blood in the intervillous space of the placenta. In cases with reduced invasion, reduced placental oxygen extraction leaves more oxygen in maternal blood (increased pO_2_ levels) and delivers less oxygen to the fetus (reduced pO_2_ levels in fetal blood).
Week 12-delivery	**Stage 1 to 2:** Alterations can only become effective with the start of maternal blood flow into the placenta, i.e. with the beginning of the second trimester	Already during the course of the first trimester, as early as 6 weeks of gestation and thus weeks before the onset of placental perfusion with maternal blood, reduced levels of syncytiotrophoblast-derived molecules have been described in maternal blood, including PP13, PAPP-A, and the fetal fraction of cell-free DNA. Such changes cannot be explained with the two-stage hypothesis.
Week 14-delivery	**Stage 2:** Placenta-derived anti-angiogenic factors are increased and are causative for the maternal symptoms	A ‘hypoxic’ placenta should secrete more pro-angiogenic factors (and not anti-angiogenic factors such as sFlt-1), to stimulate angiogenesis and to support blood flow towards the placenta. Alterations of the (anti-) angiogenic factors are only present in <20% of all preeclampsia cases; hence, such alterations cannot be responsible for the other >80% preeclampsia cases.

FGR, fetal growth restriction; PAPP-A, pregnancy-associated plasma protein-A; PE, preeclampsia; pO_2_, oxygen partial pressure; PP13, placental protein 13; sFlt-1, soluble Fms-like tyrosine kinase-1.

Therefore, it is quite interesting to see that this hypothesis is still about the same as 30 years ago, although it has already been challenged several times and several years ago (e.g. Ness and Roberts, 1996; Huppertz, 2008). The repeated criticism has led to multiple extensions of the two-stage hypothesis; however, the core statement remains the same. Today, all who are working on the human placenta and preeclampsia should be aware of the following points (Table 2) (Huppertz, 2008).

1. Shallow invasion and thus impaired transformation of uterine spiral arteries is present in about 10–20% of all preeclampsia cases, mostly the early-onset cases suffering from FGR as well (e.g. Robillard *et al.*, 2020, 2022).
2. Shallow invasion and thus impaired transformation of uterine spiral arteries is present in cases with normotensive FGR (without any symptoms of preeclampsia) as well, and thus, this feature is not specific for preeclampsia (Sibley *et al.*, 2002).
3. Shallow invasion and thus impaired transformation of spiral arteries is not followed by reduced blood supply to the placenta (Burton *et al.*, 2009). By contrast, about the same amount of maternal blood is entering the intervillous space of the placenta; however, with a massively increased velocity from 0.1 m/s in healthy widened arteries to 1–2 m/s in untransformed arteries (Burton *et al.*, 2009).
4. Placental hypoxia (i.e. reduced oxygen levels in the maternal blood circulating through the placental intervillous space) has never been measured *in vivo*. This is true for preeclampsia and FGR, especially for the early-onset subtype. By contrast, so far, only placental hyperoxia (i.e. increased oxygen levels in maternal blood circulating through the placental intervillous space) has been measured and published in cases with early-onset FGR ± preeclampsia (summarized in Nye *et al.*, 2018; Huppertz, 2023). Sibley *et al.* (2002) have used uterine vein blood behind the placenta as surrogate for intervillous space maternal blood. These authors found increased levels of pO_2_ in maternal blood, while fetal blood in the umbilical vein showed a reduced pO_2_, in line with a reduced oxygen extraction rate in such cases. Hence, due to a decrease in placental oxygen extraction, less oxygen is delivered towards the fetus, resulting in fetal hypoxia. At the same time, this decreased placental oxygen extraction from maternal blood leads to more oxygen remaining in maternal blood in the intervillous space and thus results in placental hyperoxia, if the focus is on maternal blood.
5. Placental villous tissues are already altered in the first trimester in pregnancies that go on to develop preeclampsia, prior to the onset of maternal blood flow through the placenta. This is exemplified by a reduced morphological score of placental villi including villous branching, the number of villous buds, and the presence of veins (Hannibal *et al.*, 2018). Hence, any changes of blood flow starting in the second trimester cannot be responsible for alterations of villous tissues already in the first trimester. Such changes can be further exemplified by the reduced fetal fraction of cell-free DNA (cfDNA) in maternal blood and the reduced release of the placenta-specific placenta protein 13 (PP13) already in the first trimester of pregnancy (Huppertz *et al.*, 2008; Gerson *et al.*, 2019).

### The two-stage hypothesis: further challenges and limitations

In addition to the above-mentioned explanations that already challenge the two-stage hypothesis, there are additional and substantial doubts and limitations that remain with the two-stage hypothesis. These challenges hinder the two-stage hypothesis to fully elucidate the pathophysiological pathways and the wide clinical spectrum of the syndrome.

A key limitation of the two-stage hypothesis is the missing explanation of the vast majority of cases. Early-onset preeclampsia (often associated with FGR) typically aligns with the classical narrative of shallow trophoblast invasion and a subsequent maladaptive maternal response. However, most cases (>80%) are referred to as late-onset (>34 weeks of gestation, including preterm and term cases) displaying different etiological routes, as discussed above. A recent extension of the two-stage hypothesis (Sugulle *et al.*, 2024) attempts to accommodate the late-onset form by adding ‘exceeded placental capacity’; however, also the renewed two-stage model remains with all its limitations and restrictions.

One limitation is the missing link between ‘placental hypoxia’ and the upregulation of placenta-derived anti-angiogenic factors. While placental hypoxia is only present in pregnancies at high altitude or in women with chronic anemia, it has never been measured *in vivo* in cases with preeclampsia (Nye *et al.*, 2018; Huppertz, 2023). Also, if there would be placental hypoxia, one would expect to see an upregulation of pro-angiogenic factors to counterbalance hypoxia. By contrast, the two-stage hypothesis claims that the ‘hypoxic’ placenta produces increased amounts of anti-angiogenic factors such as sFlt-1. This conflict has not been solved yet. At the same time, it is still unclear why, in a few pregnancies (i.e. early-onset preeclampsia and early-onset FGR), placental as well as maternal factors threaten the maternal angiogenic balance, while in most cases an equilibrium of angiogenic and anti-angiogenic factors is maintained despite displaying similar symptoms (e.g. term preeclampsia) (Maynard *et al.*, 2008; Huppertz, 2018b).

Another challenge is related to the statement in the hypothesis that the (anti-) angiogenic factors sFlt-1 and PlGF are responsible for the maternal symptoms of preeclampsia. Both the early-onset as well as the late-onset forms of preeclampsia are associated with the same spectrum of symptoms, with a higher rate of additional FGR in the early-onset form. At the same time, changes of sFlt-1 and PlGF are mostly predictive only for the early-onset form (Chaemsaithong *et al.*, 2022). This is not the case for most of the late-onset cases, especially term cases (Villa *et al.*, 2013). How can the hypothesis state that sFlt-1 and PlGF induce the clinical symptoms of preeclampsia in general, if these factors are only altered in about 20% of all preeclampsia cases?

The two-stage hypothesis is placenta-centric and only insufficiently addresses maternal factors known to increase the risk to develop preeclampsia, including obesity, chronic hypertension, metabolic syndrome, genetic predisposition, and preexisting vascular dysfunction (Melchiorre *et al.*, 2022). Especially, the late-onset type of preeclampsia is increasingly recognized as being driven by dysfunction of specific maternal systems, including cardiovascular and/or metabolic maladaptation (Robillard *et al.*, 2020). This may even take place in the presence of a normal early placentation and normal placental and fetal development (Huppertz, 2008), but with subsequent exhaustion of the maternal vascular system (Melchiorre *et al.*, 2022). This highlights the need for expanded models that integrate both maternal and placental health and dysregulation in a dynamic interplay (Huppertz, 2015).

Finally, recent advances have emphasized the role of placental senescence, mitochondrial dysfunction, inflammation, and cardiovascular maladaptation as drivers for the development of preeclampsia (Melchiorre *et al.*, 2022; Deer *et al.*, 2025; Nonn *et al.*, 2025). There have been attempts to revise the two-stage hypothesis to integrate the interface between these stress signals and the traditional placental hypoxia-driven two-stage model (Sugulle *et al.*, 2024). However, integrated models outside the placental hypoxia arena are urgently needed to combine placental and maternal sources for maladaptation.

In summary, the two-stage hypothesis of preeclampsia has been instrumental in conceptualizing the syndrome; however, it more and more struggles to account for the full spectrum of clinical presentations, particularly late-onset cases. This hypothesis leaves fundamental questions about primary triggers, integration of maternal routes, and reliable diagnostics unresolved. Continuous development and refinement of new models and hypotheses, incorporating placental senescence, maternal factors, and better molecular stratification, remains a pressing challenge for the field. There is the urgent need to develop new and more suited hypotheses and to reject the classical, no longer appropriate and no longer topical route of the two-stage hypothesis.

### Alternative hypotheses: not yet challenged

Recently, Jung *et al.* (2022) have given an excellent overview on the long list of putative explanations of the etiology of preeclampsia. The current list of explanations ranges from effects of viruses and bacteria to (auto-) immune and endocrine disorders to placental defects such as premature placental aging.

**Figure 1.**
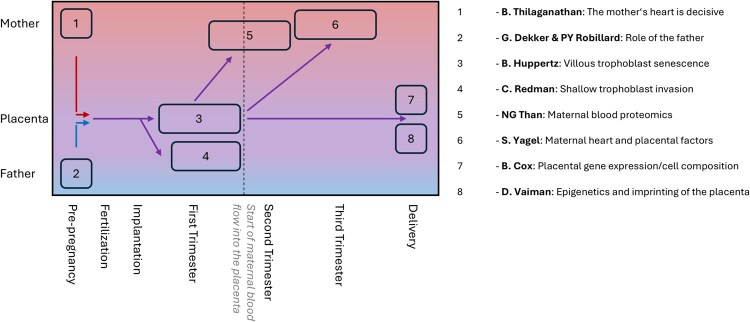
**Current hypotheses to explain the etiology of preeclampsia**. The *y*-axis shows where the hypotheses have their main focus: mother, father, placenta. The *x*-axis shows the timeline from pre-pregnancy to delivery and indicates where the hypotheses see the main effect in terms of timing or from when the samples are derived driving the hypotheses. Several groups have put forward their views and ideas to explain the broad spectrum of symptoms related to the pregnancy syndrome preeclampsia. This figure displays some of the hypotheses, accounting for only a fraction of the whole spectrum of views. (1) Thilaganathan and colleagues put the maternal heart into focus, even prior to pregnancy (Perry *et al.*, 2018), while (2) Dekker and Robillard point to the role of the father (Dekker *et al.*, 2011). (3) Huppertz describes early senescence of the villous trophoblast to be key in the etiology of preeclampsia (Huppertz, 2008, 2025), while (4) Redman and colleagues put the emphasis on shallow invasion of the extravillous trophoblast (Burton *et al.*, 2019). (5) Than *et al.* have developed clusters based on maternal blood proteomics (Than *et al.*, 2022), while (6) Yagel *et al*. combine the placental secretome with the maternal cardiovascular system (Yagel *et al.*, 2022). (7) Cox and colleagues describe clusters based on placental gene expression and cell composition (Leavey *et al.*, 2016), while (8) Vaiman and colleagues focus on placental epigenetics and imprinting (Apicella *et al.*, 2019).

Below, some of the alternative hypotheses and views are listed, representing only part of the whole spectrum of ideas (Fig. 1).

One alternative hypothesis, introduced by Basky Thilaganathan and colleagues, puts the maternal cardiovascular system into focus and states that placental factors are not needed for the development of the clinical symptoms of preeclampsia (Perry *et al.*, 2018). A maternal cardiovascular system, especially a heart, that fails to adapt to the needs of pregnancy will not pass the stress test of pregnancy and thus may end up in the syndrome preeclampsia. Pregnancy is called a stress test especially for the maternal cardiovascular system since the mother needs to adapt heart rate, stroke volume, blood volume etc to the needs of fetal growth (summarized in Brislane *et al.*, 2021). A recent meta-analysis revealed that at the time of clinical symptoms of preeclampsia, the heart mechanics show a significantly altered cardiac maladaptation to pregnancy (O’Driscoll *et al.*, 2022). It would be extremely important to reveal when such changes develop and if there are already subclinical changes prior to pregnancy.

Another attempt in creating a new explanation of the etiology of preeclampsia has been given by Yagel *et al.* (2022, 2023). These authors very nicely integrated the adaptation of the maternal heart and vascular system with the release of factors from a dysregulated placenta. Although it has been an excellent attempt to combine the pregnant woman with the placenta, these authors still use the two-stage hypothesis and placental hypoxia as basis for their new hypothesis. This definitively weakens the meaningfulness and significance of their new hypothesis.

For nearly three decades, Pierre-Yves Robillard and Gus Dekker have presented strong data on the immune adaptation of the woman to man’s semen (Dekker *et al.*, 1998). Their seminal work on the role of the father (Dekker *et al.*, 2011) has opened our eyes towards a much broader view on the pathways and individuals involved in the etiology of preeclampsia.

**Figure 2.**
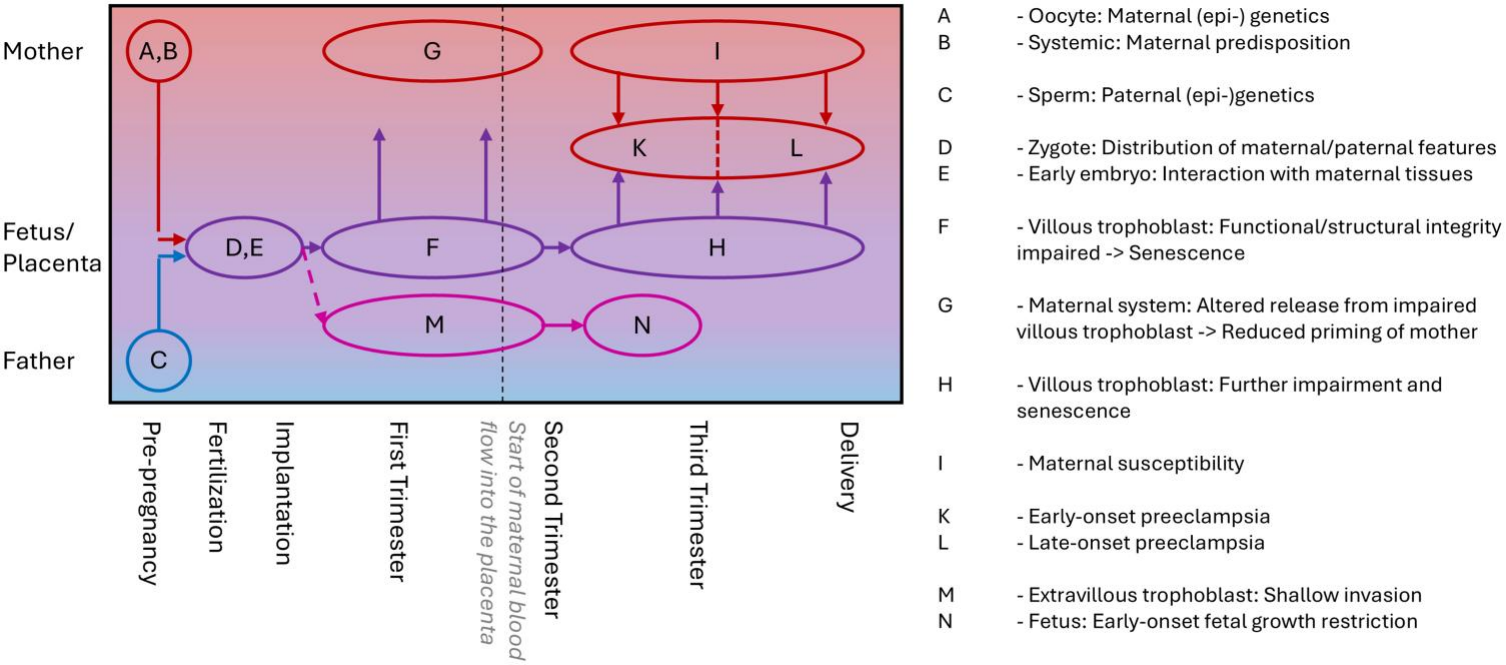
**Extended hypothesis of Huppertz**. The alternative hypothesis put forward by Huppertz (2025) tries to integrate all players involved, resulting in a multimodal explanation of the syndrome preeclampsia. The maternal and paternal germ cells may display (epi-) genetic changes even prior to pregnancy (**A**, **C**), similar to systemic predisposing features of the mother (**B**). During fertilization, maternal and/or paternal features may lead to development different from normal (**D**) or the interplay between early embryo and maternal tissues may already be defective (**E**). Based on such early changes, the villous trophoblast may show alterations on several levels (**F**) (for details see Huppertz, 2025), resulting in alterations of the placental secretome already during the first trimester of pregnancy (**G**). This leads to changes in priming the mother towards pregnancy (**G**). The further progression of villous trophoblast senescence (**H**) and the susceptibility level of the mother (**I**) define whether preeclampsia develops and requires delivery prior to 34 weeks of gestation (early-onset preeclampsia, **K**) or after 34 weeks (late-onset preeclampsia, **L**). As a different route of maldevelopment, early embryonic changes may also result in alterations of the extravillous trophoblast (**M**), subsequently causing normotensive fetal growth restriction (**N**) not related to preeclampsia.

The present author has created an alternative hypothesis, already challenging the two-stage hypothesis in 2008 (Huppertz, 2008) and has since developed a multimodal explanation series including maternal and paternal as well as placental traits, all of which contribute to the various (sub-) types of preeclampsia (Huppertz, 2011, 2015, 2018a,b). A recent approach shows that changes of the placenta in the first trimester of pregnancy can be found in all subtypes of preeclampsia (Figs 1 and 2) (Huppertz, 2025).

In addition to the trials to decipher the etiology of preeclampsia, there are a number of studies trying to subgroup the syndrome based on data on the heterogeneity of preeclampsia (Than *et al.*, 2022).

1. Brian Cox and colleagues have focused on gene expression and cell type composition in the placenta to define different subtypes of preeclampsia (Leavey *et al.*, 2016, 2019; Benton *et al.*, 2018) (Fig. 1). These authors started with the definition of five clusters, and finally reduced the number to three clusters: (a) a pure maternal origin with healthy placentas and term delivery, (b) an immunologic etiology with FGR and signs of maternal rejection of the fetus, and (c) a canonical etiology displaying FGR, early delivery, altered angiogenic status and placental dysfunction.
2. Nandor Gabor Than and colleagues (Than *et al.* 2022, 2023) have focused on data from maternal blood proteomics using longitudinal blood samples from women later developing preeclampsia (Fig. 1). These authors have identified four clusters: (a) placental preeclampsia with mostly early-onset cases and FGR, (b) metabolic preeclampsia with proinflammatory changes, less effects to the fetus and a broad spectrum of delivery timing, (c) immunological preeclampsia with immunological changes in term preeclampsia with little effects on the fetus, and (d) maternal preeclampsia with minimal changes of the proteome, with little effects on the fetus and term delivery (Than *et al.*, 2022).
3. Daniel Vaiman and colleagues have focused on epigenetic changes of the placenta in relation to the development of preeclampsia (Apicella *et al.*, 2019; Broséus *et al.*, 2022). These authors looked into DNA methylation, histone modifications, non-coding RNA (miRNA, lncRNA), and imprinting and described changes of the above in cases suffering from preeclampsia. They described a number of differentially methylated genes and miRNAs to be associated with the development of preeclampsia.

In summary, given the huge range of symptom combinations, time lines, and risk factors, the field more and more describes a multimodal development of the symptoms related to the syndrome preeclampsia.

Similar to the fact that there is not a single biomarker allowing the prediction of all preeclampsia cases, there does not seem to exist a single cause for the syndrome. Therefore, we need to subgroup the syndrome and find a cause for each subgroup or set up a multimodal explanation model with multiple possibilities to combine causes and effects. Especially the very early events are still a real mystery, as most hypotheses start with alterations at the beginning of the second trimester and ignore events in very early pregnancy or even before pregnancy (Huppertz, 2025).

## Conclusion

The two-stage hypothesis to explain the etiology of preeclampsia has reached the end of its life span and has been kept alive for no good reason. Hence, there is an urgent need for new ideas and concepts to better explain the diversity of preeclampsia in terms of symptoms and their time of occurrence during pregnancy. These can be used to subsequently develop better predictive biomarkers and treatment options.

## Data Availability

No new data were generated or analyzed in support of this review.
